# Functional Changes of Rhizosphere and Non-Rhizosphere Soils Under the Decline of *Pinus sylvestris* var. *mongolica* Plantations

**DOI:** 10.3390/plants14182819

**Published:** 2025-09-09

**Authors:** Tao Kong, Zeyu Zeng, Haotian Cheng, Sinuo Bao, Lin Xiao, Tong Liu, Xiaoliang Zhao

**Affiliations:** 1 College of Environmental Science and Engineering, Liaoning Technical University, Fuxin 123000, China; zzyxb98@163.com (Z.Z.); bsn08219819@163.com (S.B.); linx00787@gmail.com (L.X.); ltong8523@gmail.com (T.L.); xiaoliangzhao67@gmail.com (X.Z.); 2 Institute of Applied Ecology, Chinese Academy of Sciences, Shenyang 110016, China; chenghaotian@iae.ac.cn

**Keywords:** *Pinus sylvestris* var. *mongolica*, rhizosphere soils, non-rhizosphere soils, soil nutrients, stand age, microbial biomass

## Abstract

The decline of Mongolian Scots pine (*Pinus sylvestris* var. *mongolica*) plantations in the “Three-North” shelterbelt region is closely linked to soil degradation. This study compared rhizosphere and non-rhizosphere soils across different stand ages, focusing on nutrient availability, microbial biomass, enzyme activities, and soil particle morphology. Results showed that SOC and TN accumulated with age, whereas AP, AK, and pH declined in older stands, indicating progressive acidification. Results demonstrated that SOC and TN increased with stand age, whereas AP, AK, and pH exhibited a marked decline in the older stands (stands aged ≥ 40 years), reflecting progressive acidification and nutrient depletion. Rhizosphere soils consistently displayed higher SOC, TN, microbial biomass, and enzyme activities than non-rhizosphere soils, largely driven by root exudation and enhanced microbial turnover. The increasing C_mic_/N_mic_ ratio with age suggested a fungal-dominated microbial community, which may exacerbate stand decline by fostering pathogenic fungi. Scanning electron microscopy revealed pronounced particle fragmentation and surface roughness with increasing stand age, particularly in rhizosphere soils, indicating root-driven physical and biochemical weathering. These findings highlight the synergistic effects of stand development and rhizosphere processes on soil structure and fertility, providing a theoretical basis for the sustainable management and restoration of declining plantations.

## 1. Introduction

*Pinus sylvestris* var. *mongolica*, an evergreen species of the Pinaceae family, has been widely introduced and cultivated across the “Three-North” shelterbelt region due to its exceptional cold resistance, drought tolerance, and low soil requirements [1,2]. The Zhanggutai area, located on the southeastern edge of the Horqin Sandy Land, is one of the earliest regions where this species was introduced, resulting in a mosaic of stands of varying ages [3]. As a starting point for modern scientific desertification control in China, the successful afforestation and sand fixation practices using *P. sylvestris* var. *mongolica* at Zhanggutai not only improved the local ecological environment but also provided valuable experience for desertification control nationwide and globally [4]. However, since the early 1990s, artificial forests of *P. sylvestris* var. *mongolica* in Zhanggutai have experienced severe decline due to a combination of anthropogenic disturbances and natural factors [5,6]. Declining plantations exhibit a characteristic gray-green appearance, darker in tone compared to healthy stands, and similar decline phenomena have subsequently been reported in several provinces across China [5]. This large-scale decline poses significant threats to the construction of protective forests and sand management efforts in the “Three-North” shelterbelt region [7]. Therefore, investigating the decline of *P. sylvestris* var. *mongolica* plantations is critical to safeguarding ecological security, promoting ecosystem restoration, improving the economic benefits of forestry, and providing scientific evidence to support policy-making and sustainable forest management.

Soil plays a fundamental role in the functioning of forest ecosystems by driving nutrient cycling and supporting overall ecosystem productivity [8,9]. In artificial forests, soil quality directly affects stand growth and long-term sustainability, as it determines the availability of key nutrients such as carbon (C), nitrogen (N), phosphorus (P) [10], and potassium (K) [11]. With forest development and aging, dynamic changes in soil properties occur due to litter accumulation [12], root turnover, and microbial activity, which collectively regulate the balance of nutrient inputs and outputs [13]. Understanding these changes is crucial for improving soil fertility management and maintaining the productivity of *P. sylvestris* var. *mongolica* plantations.

The rhizosphere, defined as the soil zone under the direct influence of plant roots, is considered a “hotspot” of biological and chemical interactions [14]. Root exudation [15], microbial metabolism [16,17], and redox processes lead to significant differences in the physicochemical properties, microbial community structure, and nutrient cycling processes between rhizosphere and non-rhizosphere soils [18]. Previous studies have shown that rhizosphere effects can significantly alter organic carbon turnover, the availability of nitrogen and phosphorus, and soil enzyme activity [19], all of which directly influence plant nutrient uptake and soil fertility maintenance [20].

In plantation ecosystems, stand age [21] is a key factor in differences between regulating rhizosphere and non-rhizosphere soil. During the development of a forest, the differences in litter decomposition [22] and root vitality create unique stage patterns of soil nutrient dynamics [23]. However, studies examining the interactive changes in rhizosphere and non-rhizosphere soil nutrients, microbial biomass, and enzyme activities across stand ages in *P. sylvestris* var. *mongolica* plantations remain limited. Moreover, soil particle morphology and microstructure, which play vital roles in nutrient retention and microbial habitat formation [9], have not been directly compared between rhizosphere and non-rhizosphere soils. Scanning electron microscopy (SEM) provides high-resolution visualization of soil particle morphology and microstructural characteristics, offering critical insights into nutrient retention mechanisms and microbial habitat formation [24]. Therefore, this study investigates the nutrient dynamics, microbial biomass, enzyme activities, and microstructural characteristics of rhizosphere and non-rhizosphere soils across different stand ages of *P. sylvestris* var. *mongolica*, aiming to elucidate the combined effects of stand age and rhizosphere processes on soil ecological functioning. Our findings provide theoretical support for soil quality assessment and the sustainable management of artificial plantations.

## 2. Results

### 2.1. Nutrient Content in Rhizosphere and Non-Rhizosphere Soils of Pinus sylvestris var. mongolica Plantations at Different Stand Ages

**Figure 1:**
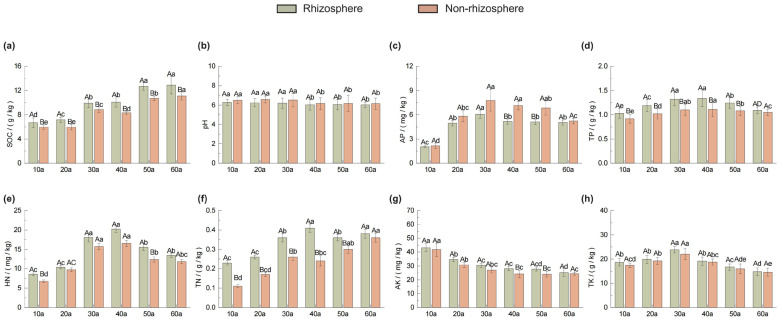
Nutrient content of rhizosphere and non-rhizosphere soils of *Pinus sylvestris* var. *mongolica* at different stand ages (10a–60a, years). (**a**) SOC: soil organic carbon; (**b**) pH; (**c**) AP: available phosphorus; (**d**) TP: total phosphorus; (**e**) HN: alkali-hydrolyzable nitrogen; (**f**) TN: total nitrogen; (**g**) AK: available potassium; (**h**) TK: total potassium. Values are means + standard deviations (*n* = 3). Capital letters denote statistically significant differences between rhizosphere and non-rhizosphere soils at the same sampling location and stand age (*p* < 0.05), while lower-case letters denote statistically significant differences among soil samples taken from the same position across different stand ages (*p* < 0.05).

This study (Figure 1) demonstrates that the nutrient content of the rhizosphere and non-rhizosphere soils of the *Pinus sylvestris* var. *mongolica* Plantations exhibits regular changes with stand age: soil organic carbon (SOC) continues to increase, reaching its peak at 60a, SOC in the rhizosphere is consistently significantly higher than that in the non-rhizosphere (*p* < 0.05) (Figure 1a); total nitrogen (TN) in the rhizosphere peaks at 40a and then declines, while in the non-rhizosphere it continues to rise until 60a (Figure 1f). Available phosphorus (AP), total phosphorus (TP), alkaline nitrogen (HN) and total potassium (TK) show a unimodal distribution (HN, AP and TK at 30a). Available potassium (AK) and pH decline with stand age and stabilize after 40a (Figure 1b,g). Total nutrients significantly dominating the rhizosphere between 10 and 50a, while the difference disappears at 60a. Significant differences in available nutrients are observed between 40 and 50a (*p* < 0.05). AP and pH are consistently significantly higher in the non-rhizosphere compared to the rhizosphere (*p* < 0.05), revealing a persistent deficiency of an AP in the rhizosphere and a dynamic acidic pH characteristic caused by root exudates. Overall, nutrient heterogeneity between rhizosphere and non-rhizosphere soils diminished with stand maturation.

### 2.2. Variations in Microbial Biomass and Enzyme Activities in Rhizosphere and Non-Rhizosphere Soils Across Stand Ages

**Figure 2:**
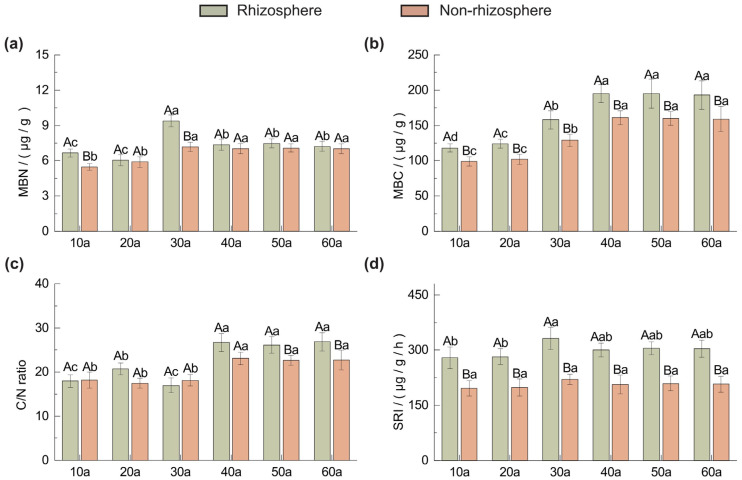
Changes in microbial biomass nitrogen (MBN) (**a**), microbial biomass carbon (MBC) (**b**), microbial biomass carbon to nitrogen ratio (C/N ratio) (**c**), and soil respiration intensity (SRI) (**d**) in rhizosphere and non-rhizosphere soils of different stand ages (10a–60a, years). Values are means + standard deviations (*n* = 3). Capital letters denote statistically significant differences between rhizosphere and non-rhizosphere soils at the same sampling location and stand age (*p* < 0.05), while lower-case letters denote statistically significant differences among soil samples taken from the same position across different stand ages (*p* < 0.05).

As shown in Figure 2, microbial biomass carbon (MBC) (Figure 2b) and the microbial biomass C:N ratio (Figure 2c) in both rhizosphere and non-rhizosphere soils of *Pinus sylvestris* var. *mongolica* plantations increased initially with stand age and then stabilized, whereas microbial biomass nitrogen (MBN) (Figure 2a) and soil respiration exhibited a pattern of increase followed by decline and stabilization. Across all ages, rhizosphere soils had higher MBC, MBN, soil respiration, and C:N ratios than non-rhizosphere soils. Significant differences (*p <* 0.05) were observed for MBC and soil respiration at all ages (Figure 2b), for MBN at 10a and 30a, and for the microbial biomass C:N ratio at 50a and 60a (*p* < 0.05) (Figure 2c).

**Figure 3:**
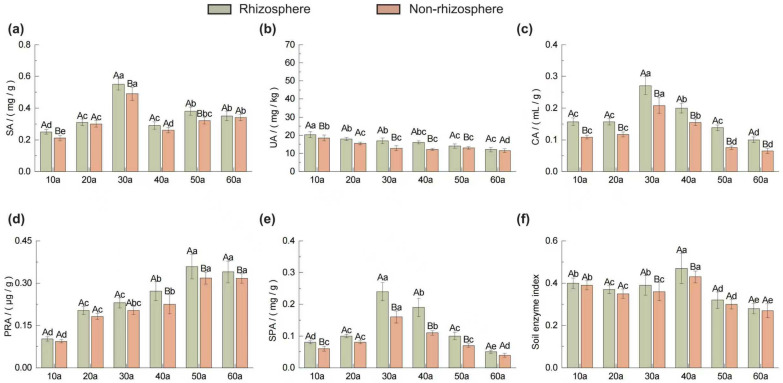
Soil enzyme activity in rhizosphere and non-rhizosphere soils of *Pinus sylvestris* var. *mongolica* at different stand ages (10a–60a, years). (**a**) SA: sucrase activity, (**b**) UA: urease activity, (**c**) CA: catalase activity, (**d**) PRA: protease activity (**e**) SPA: soil phosphatase activity and (**f**) Soil enzyme index. Values are means + standard deviations (*n* = 3). Capital letters denote statistically significant differences between rhizosphere and non-rhizosphere soils at the same sampling location and stand age (*p* < 0.05), while lower-case letters denote statistically significant differences among soil samples taken from the same position across different stand ages (*p* < 0.05).

As shown in Figure 3, rhizosphere and non-rhizosphere soils of *Pinus sylvestris* var. *mongolica* plantations exhibited distinct patterns of enzyme activity with increasing stand age. Sucrase activity (SA) increased initially, peaked at 30a, and then declined, whereas urease activity (UA) and protease activity (PRA) showed opposite trends of gradual decrease and increase, respectively (Figure 3a,b,d). Phosphatase activity (SPA) and catalase activity (CA) displayed unimodal patterns with maxima also at 30a (Figure 3c,e). Rhizosphere enzyme activities were generally higher than non-rhizosphere values, with significant differences observed for most enzymes across ages (*p* < 0.05).

The Soil Enzyme Index (SEI) (Figure 3f), used to integrate multiple enzyme activities, revealed a “stable–increase–decrease” trend with stand age, peaking at 40a and reaching the lowest value at 60a. SEI was consistently higher in rhizosphere than in non-rhizosphere soils, with the greatest difference at 40a; significant differences occurred at 30a and 40a (*p* < 0.05).

### 2.3. Dynamics of Rhizosphere Effects Across Stand Ages

**Figure 4:**
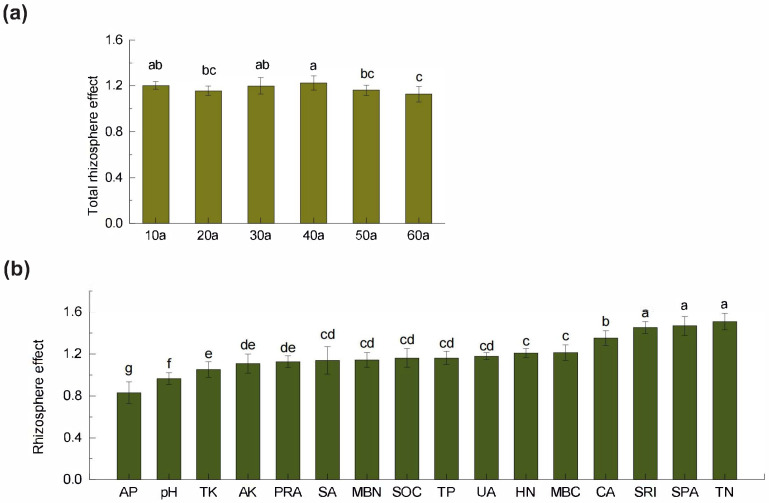
(**a**) Total rhizosphere effect of soil index in *Pinus sylvestris* var. *mongolica* plantations at different stand ages (10a–60a, years). (**b**) Rhizosphere effect of soil property index in *Pinus sylvestris* var. *mongolica* plantations. AP: available phosphorus; TK: total potassium; AK: available potassium; PRA: protease activity; SA: sucrase activity; MBN: microbial biomass nitrogen; SOC: soil organic carbon; TP: total phosphorus; UA: urease activity; HN: alkali-hydrolyzable nitrogen; MBC: microbial biomass carbon; CA: catalase activity; SRI: soil respiration intensity; SPA: soil phosphatase activity and TN: total nitrogen. Values are means + standard deviations (*n* = 3). Different lowercase letters above bars indicate significant differences among treatments (*p* < 0.05).

Root activity and associated microbial processes create pronounced differences between rhizosphere and non-rhizosphere soils, with the intensity of these rhizosphere effects varying by stand age. Analysis showed (Figure 4b) that most soil nutrients—SOC, TN, HN, TP, TK, and AK—exhibited positive rhizosphere effects (RE > 1), whereas AP and pH showed negative effects (RE < 1). Among nutrients, TN had the highest RE, while SPA showed the greatest RE among microbial indicators, all of which were positive (RE > 1). Across stand ages (Figure 4a), the overall rhizosphere effects followed a “decrease–increase–decrease” pattern, peaking at 40a and reaching the minimum in overmature stands (60a).

### 2.4. Linkages Between Soil Nutrients and Microbial Indicators Revealed by RDA and Clustering

**Figure 5:**
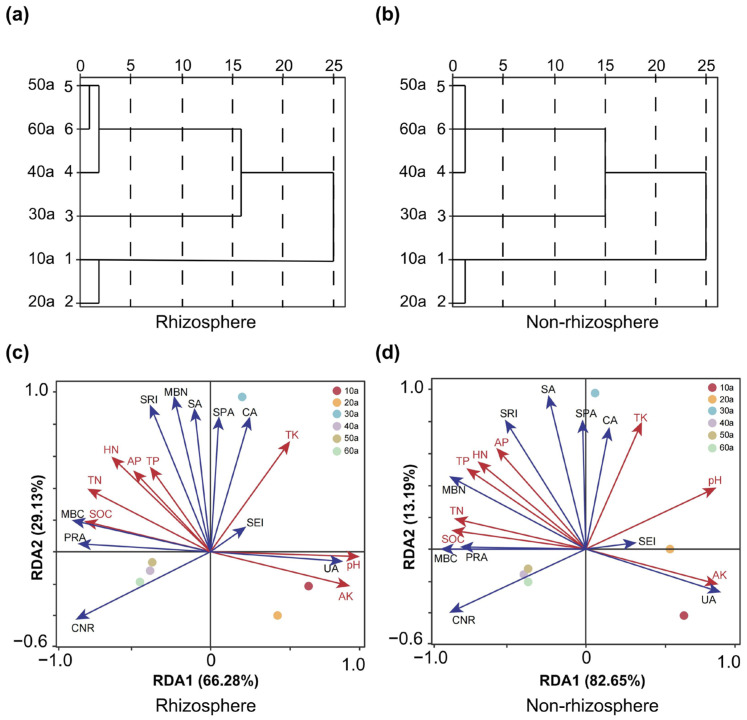
Tree diagram of cluster analysis of microbial indicators of rhizosphere (**a**) and non-rhizosphere (**b**). Redundancy analysis of soil nutrients and microbial indicators of rhizosphere (**c**) and non-rhizosphere (**d**). AP: available phosphorus; TK: total potassium; AK: available potassium; PRA: protease activity; SA: sucrase activity; MBN: microbial biomass nitrogen; SOC: soil organic carbon; TP: total phosphorus; UA: urease activity; HN: alkali-hydrolyzable nitrogen; MBC: microbial biomass carbon; CA: catalase activity; SRI: soil respiration intensity; SPA: soil phosphatase activity and TN: total nitrogen. Dots of different colors represent different stand ages; the length of the arrowed lines indicates the extent to which a nutrient factor affects microbial indicators, with longer lines signifying greater impact and shorter lines indicating lesser impact.

Using a systematic clustering analysis method, we clustered the rhizosphere and non-rhizosphere soils based on 10 microbial indicators (SA, SPA, CA, UA, PRA, SEI, MBC MBN, C/N ratio, and SRI). The clustering results for rhizosphere soils are presented in plot (Figure 5a), which can be divided into four categories: the first category is young forests (10a and 20a), the second category is middle-aged forests (30a), the third category is near-mature forests (40a), and the fourth category is mature forests (50a) and over-mature forests (60a). The clustering results for non-rhizosphere soils are presented in plot (Figure 5b), where the results are also divided into four categories: the first category is young forests (10a and 20a), the second category is middle-aged forests (30a), and the third category includes near-mature forests (40a), mature forests (50a), and over-mature forests (60a).

Redundancy analysis (RDA) (Figure 5) revealed that soil nutrients strongly influenced microbial indicators in both rhizosphere and non-rhizosphere soils of *Pinus sylvestris* var. *mongolica* plantations (Figure 5c,d). For rhizosphere soils, the first two axes explained 95.41% of total variation (axis 1: 66.28%; axis 2: 29.13%), with stand ages 40–60a clustering on the left of axis 1 and younger stands (10–30a) on the right. SOC, TN, AK, and pH were the main drivers (P_SOC_ = 0.002; P_TN_ = 0.016; P_AK_ = 0.004; P_pH_ = 0.008). For non-rhizosphere soils, axes 1 and 2 explained 95.84% of total variation (axis 1: 82.65%). Similar patterns of separation by stand age were observed. Key drivers were SOC, TN, AK, and pH (P_SOC_ = 0.042; P_TN_ = 0.038; P_AK_ = 0.002; P_pH_ = 0.030), with correlation patterns consistent with those in rhizosphere soils. RDA further indicated clear distinctions between rhizosphere and non-rhizosphere microbial–nutrient associations.

### 2.5. Characteristics of Soil Particle Morphology Changes with Tree Age

**Figure 6:**
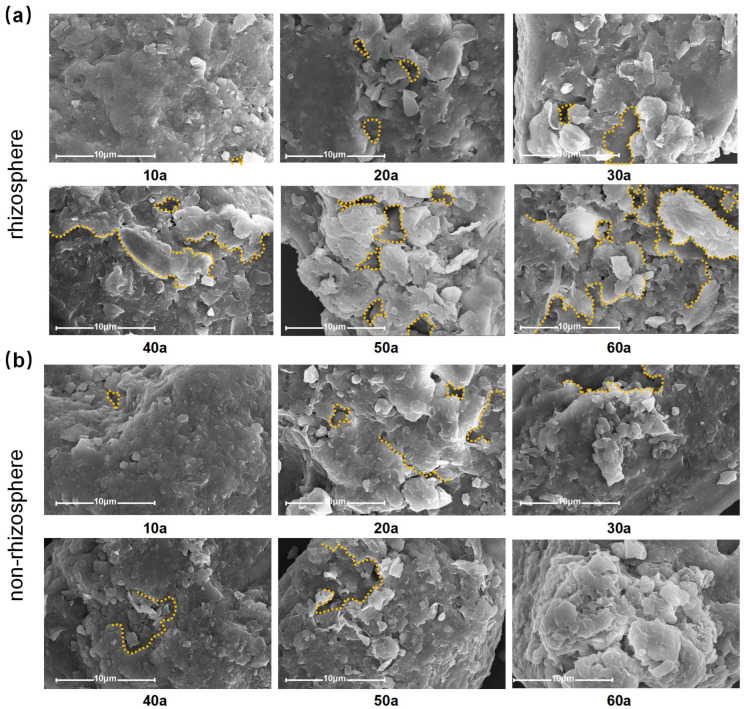
(**a**) The rhizosphere soil structure of different stand ages (10a–60a, years) under the scanning electron microscope. (**b**) The non-rhizosphere soil structure of different stand ages (10a–60a, years) under the scanning electron microscope.

Soil particle morphology in both rhizosphere and non-rhizosphere soils exhibited progressive changes with increasing stand age (Figure 6). SEM observations at 10 µm resolution revealed that young-stand soils had well-structured, intact particles, whereas older stands showed increased surface roughness, fissures, and a looser arrangement of fine skeletal particles. These changes, in addition to natural physical weathering and erosion, indicate that biological processes such as root pressure, stretching, and enzymatic action accelerate particle disintegration and structural heterogeneity with forest development.

## 3. Discussion

### 3.1. Rhizosphere Differences in Soil Nutrients Across Different Stand Ages

This study revealed that soil nutrient dynamics in *Pinus sylvestris* var. *mongolica* plantations exhibit distinct stage-specific patterns with stand age, and that nutrient levels are generally higher in rhizosphere soils than in non-rhizosphere soils (Figure 1). Both rhizosphere and non-rhizosphere SOC contents increased continuously with stand age, likely due to the thickening of litter layers and the consequent accumulation of organic carbon through decomposition and transformation processes [25,26]. TN, HN, TP, and AP increased until the near-mature stage (40a), after which they declined. This trend can be attributed to the balance between nutrient input from litter decomposition and nutrient uptake by trees: prior to 40a, the release of N and P from litter decomposition exceeded the plant demand [27], while beyond 40a, increased understory biomass [28] and reduced microbial activity decreased nutrient turnover, leading to lower N and P availability. TK and AK were lower after 40a due to intensified plant uptake, as K is mainly supplied by the soil [29]. Soil pH declined continuously with stand age, which is closely linked to the accumulation of humic substances—particularly humic acids—leading to soil acidification.

When comparing rhizosphere and non-rhizosphere soils, SOC, TN, HN, TP, TK, and AK were consistently higher in the rhizosphere, driven by root exudates that promote the accumulation of organic C and N and enhance P availability [20]. The relatively low rhizosphere pH, coupled with higher microbial biomass and enzyme activity, enhances the mobilization of N and K, explaining the elevated HN and AK contents [30]. The consistently lower AP and pH values in the rhizosphere agree with previous findings by Cheng et al. [31]. Root-induced acidification and intense nutrient uptake likely contribute to a negative rhizosphere effect on AP [32], as organic acid activation of P is weaker than root uptake, leading to P depletion near the root surface.

### 3.2. Soil Microbial Biomass and Soil Respiration

Our findings indicate that both MBC and MBN showing a “rise–fall” trend with stand age (Figure 2). As key indicators of soil quality and fertility [33,34], MBC and MBN were enhanced in the rhizosphere due to root exudates providing easily available C and N substrates, which fostered favorable conditions for microbial proliferation [35,36]. Soil respiration can also explain this phenomenon. Soil respiration is a key pathway in the carbon cycle of forest ecosystems [37]. Microbial respiration typically accounts for over 60% of total soil respiration, while root and litter respiration account for approximately 20% and 10%, respectively [38]. In this study, the intensity of soil respiration under different stand ages was significantly positively correlated with microbial biomass carbon, indicating that microbial activity is the primary driving factor of soil respiration. The peak in MBC and MBN at 30a suggests a maximum root exudation rate in mid-aged stands, with subsequent declines likely linked to reduced root vitality and substrate availability.

The soil microbial biomass carbon to nitrogen ratio reflects the proportion of bacteria to fungi in the soil [39]. Since the C/N ratio of fungi is higher than that of bacteria, C_mic_/N_mic_ is typically used as an indicator of changes in microbial communities [40]. Specifically, elevated C_mic_/N_mic_ may reflect a fungal-dominated community, though molecular data are needed for verification [41]. This study found that the C_mic_/N_mic_ ratio increased with stand age, whereas soil pH decreased progressively. This pattern may result from soil acidification caused by the thickening litter layer, which favors fungi due to their greater tolerance to acidic environments, thereby increasing the fungal proportion in microbial communities [42]. The C_mic_/N_mic_ ratio was consistently higher in rhizosphere soils than in non-rhizosphere soils, indicating that the rhizosphere provides a more favorable environment for fungal growth. Future studies are recommended to examine and elucidate the differences and trends in microbial community structure between rhizosphere and non-rhizosphere soils across different stand ages, in order to uncover the underlying causes of their decline from the perspective of microbial community dynamics.

### 3.3. Soil Enzyme Activities

This study demonstrates significant variation in soil enzyme activities with stand age, with rhizosphere soils exhibiting consistently higher activities compared to non-rhizosphere soils (Figure 3). Sucrase activity (SA) peaked at 30a, reflecting enhanced organic matter turnover and improved soil aeration during this stage [43]. Urease activity (UA) declined with age due to progressive urea hydrolysis and N assimilation by plants, reducing the available substrate [44]. Catalase activity (CA) showed a peak at 30a, likely due to increased generation of hydrogen peroxide during organic matter decomposition, which induced higher enzyme activity [45]. Phosphatase activity (SPA) exhibited its highest activity in mid-aged stands and declined thereafter, consistent with the reduced turnover of organic P and the decrease in available P [46]. Protease activity (PRA) increased with total N accumulation, peaking at 50a, suggesting highly active N cycling in mature stands. The consistently higher enzyme activities in the rhizosphere are attributed to root exudation of low-molecular-weight organic compounds, which provide substrates for enzymatic reactions and stimulate microbial activity [47].

### 3.4. Soil Particle Morphology

Our results show that soil particle morphology undergoes marked alterations with stand age, with more pronounced changes in rhizosphere soils. Scanning electron microscopy (SEM) images (Figure 6) revealed that young stands (10a) had compact and well-structured particles, while older stands exhibited more pronounced fissures, increased surface roughness, and a looser arrangement of fine skeletal particles. These changes were particularly evident in rhizosphere soils, likely due to mechanical stresses (compression and stretching) exerted by roots, as well as enzymatic degradation and dissolution caused by microbial activity [47,48]. Additionally, chemical processes such as acidification and nutrient depletion may have further contributed to the irregular and fragmented morphology of soil particles with increasing stand age [49]. These findings suggest that stand development and rhizosphere processes jointly accelerate the physical and chemical weathering of soil particles, which may have implications for soil structure stability and nutrient dynamics.

## 4. Materials and Methods

### 4.1. Study Area

**Figure 7:**
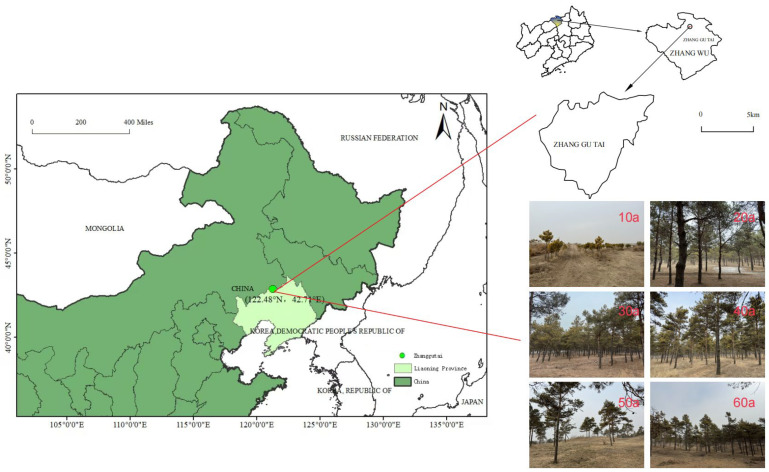
Location of study area in Zhanggutai Town, Zhangwu County, Liaoning Province, China. Site conditions of *Pinus sylvestris* var. *mongolica* plantations of different ages (10a–60a, years).

This study is located at the afforestation experimental base in Zhanggutai Town, Zhangwu County, Liaoning Province, China (42°43′–42°51′ N, 121°53′–122°22′ E), situated on the southeastern edge of the Horqin Sandy Land (Figure 7) (Table 1). It is one of the earliest regions in China to conduct introduction trials of Mongolian Scots pine (*Pinus sylvestris* var. *mongolica*) and is also an important component of the "Three-North" Shelter Forest Program. The area has a temperate sub-humid continental monsoon climate, with an average annual temperature of approximately 5.5 °C, a January average temperature of −16.3 °C, and a July average temperature of 23.9 °C. The average annual precipitation is between 450 and 550 mm, with about 70% of the rainfall concentrated in the summer months from June to August. The annual evaporation rate reaches 1300 to 1800 mm, indicating a relatively high degree of aridity. The average annual wind speed is 4.5 m·s^−1^, with stronger winds observed in spring and winter, reaching a maximum wind speed of up to 5.0 m·s^−1^. The soil type in this region is primarily sandy soil, followed by silt, with the lowest proportion of clay, and surface vegetation is dominated by drought-tolerant xerophytes. The dominant plant species include Mongolian Scots pine (*Pinus sylvestris* var. *mongolica*), saltbush (*Artemisia halodendron*), Gordejev willow (*Salix gordejevii*), wild reed (*Arundinella anomala*), and small white mugwort (*Artemisia frigida*), which exhibit typical ecological characteristics of arid zone vegetation.

### 4.2. Plot Selection and Sample Collection

After conducting a thorough investigation of *Pinus sylvestris* var. *mongolica* plantations in the study area, sample plots with consistent site conditions across different stand ages (10a, 20a, 30a, 40a, 50a, and 60a) were selected, ensuring that all plots were established on fixed sandy soil prior to the creation of the Larix gmelinii artificial forest. Within each stand age plot, five standard plots measuring 20 m × 20 m were established for five repetitions. The basic conditions of the plots are summarized in Table 1. In each standard plot, five representative trees were selected to collect soil samples from both rhizosphere and non-rhizosphere areas. For each representative tree, four soil samples were taken from the 0–40 cm soil layer in the east, south, west, and north directions [32]. The 20 soil samples were then mixed together to serve as the soil sample for the assessment of that standard plot.

To investigate the impact of plant rhizosphere effects on soil properties, representative plants were randomly selected within the sample plots. Soil samples from both the rhizosphere and non-rhizosphere soil were collected by vertically profiling along the root direction and stratifying with a spade. Rhizosphere soil was obtained by gently tapping the root surface to collect soil tightly adhering to the roots within a range of approximately 1~2 mm. Non-rhizosphere soil, serving as the control, was collected from soil located more than 5 cm away from the roots [2]. All samples were immediately sieved through a 2 mm mesh to remove impurities upon returning to the laboratory. A portion of the soil samples was refrigerated for the determination of soil enzyme activity and microbial biomass, while another portion of the soil samples, requiring nutrient analysis, was air-dried.

### 4.3. Soil Properties and Enzyme Activity

Soil pH was determined using the potentiometric method [50]. Soil organic carbon (SOC) was measured through dichromate oxidation followed by ferrous sulfate titration. Total nitrogen (TN) was assessed using the Kjeldahl digestion and distillation method. Alkali-hydrolyzable nitrogen (HN) was determined through the alkaline diffusion method [51]. Total phosphorus (TP) and available phosphorus (AP) in the soil were quantified using the molybdenum-antimony colorimetric method. Total potassium (TK) and available potassium (AK) in the soil [11] were determined by flame photometry following digestion with HF-HClO_4_. Soil microbial biomass nitrogen was determined using the Kjeldahl digestion-distillation method, and soil microbial biomass carbon was measured by the chloroform fumigation-extraction method. Soil basal respiration intensity was assessed using the static alkali absorption method [52].

Soil particle morphology and pore structure were examined using a scanning electron microscope (SEM; Apreo S, Thermo Fisher Scientific, Waltham, MA, USA). Before the test, air-dry the soil samples. Each powder sample requires 10 mg. Air-dried soil samples were carefully mounted on aluminum stubs using conductive double-sided carbon tape. The samples were then coated with a thin layer of gold using a vacuum sputter coater to enhance conductivity and imaging quality. SEM observations were performed under an accelerating voltage of 30 kV, finding three scanning points, and representative micrographs were captured to characterize the surface morphology and microstructural features of rhizosphere and non-rhizosphere soils [53,54].

The determination of soil phosphatase activity (Phosphatase activity, SPA) was conducted using the p-nitrophenyl phosphate method. The measurement of soil sucrase activity (Sucrase activity, SA) was performed using the dinitrosalicylic acid colorimetric method. The assessment of soil urease activity (Urease activity, UA) was carried out using the sodium hypochlorite colorimetric method [44]. The evaluation of soil catalase activity (Catalase activity, CA) was conducted using the titration method. Finally, the determination of soil protease activity (Protease activity, PRA) was performed using the ninhydrin colorimetric method [55,56]. The Soil Enzymes Index (SEI) is calculated based on the measurement of enzyme activity [57], and the formula is as follows [58]:

(1) $$\;S_{i}=\;\frac{x_{i}-x_{\;min}}{x_{\;max}-x_{min}}$$

where *S_i_* signifies the score for the *i*-th indicator, *x_i_* represents the measured value, and *x_max_* and *x_min_* denote the maximum and minimum values, respectively.

In the second phase, the weight (*W_i_*) assigned to each indicator was calculated through a principal component analysis, which involved dividing the variance of its shared factors by the overall variance of all indicators [58].

(2) $$\;W_{i}=\frac{C_{i}}{C}$$

The SEI was calculated by multiplying the assigned values by their respective weights. Increased values of SEI suggest a rise in both soil quality and enzyme activity.

(3) $$\;SEI=\sum_{i=1}^{n}W_{i}\times S_{i}$$

In this formula, *n* denotes the total number of indicators, while *S_i_* and *W_i_* represent the score and weight of the *i*-th indicator [58].

### 4.4. Statistical Analysis

Statistical analyses were performed using SPSS 20.0 software, with one-way analysis of variance (ANOVA) followed by Duncan’s multiple range test. Graphical representations of the data were generated using OriginPro 2024 software. The rhizosphere effect (RE) was calculated to quantify the influence of root-associated processes on soil nutrients and microbial indicators. Rhizosphere effect (RE) was calculated as follows:

(4) $$\;RE\;=\;\frac{VR}{VB}$$

VR denotes rhizosphere metric data, whereas VB refers to non-rhizosphere metric data. RE > 1 indicates a positive rhizosphere effect, whereas RE < 1 indicates a negative effect. Hierarchical cluster analysis (HCA) was applied to group different stand ages based on 10 microbial indicators (SA, SPA, CA, UA, PRA, SEI, MBC, MBN, C/N ratio, and SRI). The Euclidean distance matrix and Ward’s minimum variance method were employed to generate the clustering dendrograms, which were visualized using the ‘pheatmap’ package in R (version 4.5; R Core Team, 2025).

Redundancy analysis (RDA) was conducted to examine the relationships between soil microbial indicators and soil physicochemical properties (SOC, TN, HN, TP, AP, TK, AK, and pH) across different stand ages. The significance of environmental variables was tested using the Monte Carlo permutation test (999 permutations). RDA ordination plots were generated using the ‘vegan’ package in R (version 4.5; R Core Team, 2025), and the ‘ggplot2’ package was employed to create figures that visualize results.

## 5. Conclusions

This study highlights the significant changes in soil physicochemical properties, microbial activity, and particle morphology in *Pinus sylvestris* var. *mongolica* plantations with stand age and rhizosphere effects. SOC increased continuously with stand age, while TN, HN, TP, and AP peaked at 40 years and then declined, reflecting a dynamic balance between litter decomposition and plant nutrient uptake. Nutrient levels were generally higher in rhizosphere soils due to root exudates enhancing the mobilization of C, N, and K, whereas AP and pH showed negative rhizosphere effects. Both MBC and MBN exhibited a “rise–fall” trend, peaking at 30 years, while increasing C_mic_/N_mic_ and decreasing pH indicated a shift toward fungal dominance. Soil enzyme activities showed stage-specific variations, with consistently higher activity in the rhizosphere. SEM observations revealed progressive fragmentation and roughening of soil particles with stand age, particularly in rhizosphere soils. These findings suggest that stand development and rhizosphere processes jointly drive nutrient cycling and soil structural evolution, which are critical for sustaining plantation health. The decline of *Pinus sylvestris* var. *mongolica* plantations appears to be driven by weakened rhizosphere enzyme activity and disrupted soil nutrient balance across different stand ages. These findings may provide guidance for the prevention and remediation of decline in *Pinus sylvestris* var. *mongolica* plantations as well as other shelterbelt forests in the “Three North” region.

## Figures and Tables

**Table 1:** Basic information of sample plots.

Site No.	Stand Age/a	Mean Canopy Height/m	Mean Diameter/cm	Average Crown width (East, West)/m	Average Crown Width (South, North)/m	Soil Bulk Density/(g·cm^−3^)
1	10a	3.28	7.50	2.59	2.42	1.67
2	20a	7.80	13.31	3.60	4.21	1.64
3	30a	9.25	16.69	4.01	4.10	1.60
4	40a	11.27	20.92	4.30	4.69	1.56
5	50a	11.01	19.61	4.59	4.72	1.60
6	60a	13.04	20.59	4.62	4.92	1.62

## Data Availability

The original contributions presented in this study are included in the article. Further inquiries can be directed to the corresponding authors.
